# Identification of Four-Jointed Box 1 (FJX1)-Specific Peptides for Immunotherapy of Nasopharyngeal Carcinoma

**DOI:** 10.1371/journal.pone.0130464

**Published:** 2015-11-04

**Authors:** San Jiun Chai, Yoke Yeow Yap, Yoke Ching Foo, Lee Fah Yap, Sathibalan Ponniah, Soo Hwang Teo, Sok Ching Cheong, Vyomesh Patel, Kue Peng Lim

**Affiliations:** 1 Cancer Research Initiatives Foundation (CARIF), Sime Darby Medical Centre, Subang Jaya, Selangor, Malaysia; 2 Department of Surgery, Clinical Campus Faculty of Medicine and Health Sciences, Universiti Putra Malaysia, Hospital Kuala Lumpur, Kuala Lumpur, Malaysia; 3 Department of Oncology, Sime Darby Medical Centre, Subang Jaya, Selangor, Malaysia; 4 Department of Surgery, Cancer Vaccine Development Program, Uniformed Services University of the Health Sciences, Bethesda, Maryland, United States of America; University of Nebraska - Lincoln, UNITED STATES

## Abstract

Nasopharyngeal carcinoma (NPC) is highly prevalent in South East Asia and China. The poor outcome is due to late presentation, recurrence, distant metastasis and limited therapeutic options. For improved treatment outcome, immunotherapeutic approaches focusing on dendritic and autologous cytotoxic T-cell based therapies have been developed, but cost and infrastructure remain barriers for implementing these in low-resource settings. As our prior observations had found that four-jointed box 1 (FJX1), a tumor antigen, is overexpressed in NPCs, we investigated if short 9–20 amino acid sequence specific peptides matching to FJX1 requiring only intramuscular immunization to train host immune systems would be a better treatment option for this disease. Thus, we designed 8 FJX1-specific peptides and implemented an assay system to first, assess the binding of these peptides to HLA-A2 molecules on T2 cells. After, ELISPOT assays were used to determine the peptides immunogenicity and ability to induce potential cytotoxicity activity towards cancer cells. Also, T-cell proliferation assay was used to evaluate the potential of MHC class II peptides to stimulate the expansion of isolated T-cells. Our results demonstrate that these peptides are immunogenic and peptide stimulated T-cells were able to induce peptide-specific cytolytic activity specifically against FJX1-expressing cancer cells. In addition, we demonstrated that the MHC class II peptides were capable of inducing T-cell proliferation. Our results suggest that these peptides are capable of inducing specific cytotoxic cytokines secretion against FJX1-expressing cancer cells and serve as a potential vaccine-based therapy for NPC patients.

## Introduction

Nasopharyngeal carcinoma (NPC) is a malignant tumor of the nasopharyngeal epithelium, which is widely known for its peculiarly skewed worldwide incidence. This disease is largely prevalent in South East Asia, where approximately 70,000 new cases and 41,000 deaths were recorded for 2012 [1–3]. In Malaysia overall, NPC represents the forth most prevalent cancer and the third most common cancer amongst men [4]. However, among the Bidayuh indigenous population of Sarawak (East Malaysia), NPC incidence rates are the highest when compared to other cancers and this is an example of a regional hotspot that adds to the skewedness of this disease [5].

At early stages of the disease, NPC patients generally respond well to chemo/radiotherapy, and with intensity-modulated radiotherapy (IMRT), the loco-regional control of early stage NPC can exceed 91% [6–7]. However, treatment cost and the availability of IMRT facilities in rural and remote areas are the major challenges in managing NPC, especially for socioeconomic disadvantaged groups in developing countries where NPC is endemic. This is further confounded by the fact that NPC is typically diagnosed at late stages and up to 58% will suffer from disease recurrence within 2 years, adversely impacting NPC patient survival [8–11]. Noteworthy, recurrences are more aggressive generally by inflicting damage to surrounding tissues, for example nerves and vital organs. Treatment options for these advance lesions become limited [12]. Although chemotherapy is used for recurrent NPCs, response rates are ~ 65% with a mean survival of less than 1 year [13]. Hence, managing recurrent NPC remains a clinical dilemma and represents an urgent and unmet need to develop tumor specific treatments and novel approaches of preventing recurrence for NPC patients to help improve outcome [10].

Cancer immunotherapy, an approach to harness cancer patients’ immune system to recognize and eradicate tumor cells, recently described as one of the “breakthrough’s of 2013” in light of favorable outcomes of several clinical trials, has emerged as a potential cancer treatment modality [14]. As an example, peptide vaccine designed against Her2/neu (nelipepimut-S) and LY6K showed promising results in phase II clinical trials [15–16]. Given the strong etiological link between Epstein-Barr virus (EBV) and NPC, EBV-encoded proteins such as LMP1, LMP2 and EBNA1 have been used as vaccine targets for NPC. While, enhanced immunity and sustained clinical responses in NPC patients have been reported [17–19], EBV-encoded proteins such as LMP1 and LMP2 are weakly immunogenic [20] and frequently evolve to acquire mutations within the A2-restricted epitope [21] resulting in either a poor antigen presentation or poor T-cell priming. Similarly, a modified vaccinia virus encoding a functionally inactive fusion protein of LMP2 and EBNA1 recently evaluated in a phase I clinical trial, was found to be well tolerated but efficacy is yet to be tested [22]. On the other hand, adoptive immunotherapy for the treatment of metastatic NPC has been explored in a phase I clinical trial, albeit with mixed outcome. While the adoptive transfer of autologous cytotoxic T-lymphocytes specific for LMP was well tolerated, the treatment was only effective at regressing metastatic lesions and not the primary tumor [23]. Thus, additional and efficient tumor targets are needed for developing immunotherapies for treating NPC.

From our recently conducted mRNA expression analysis of clinically defined cohort of NPC specimens, we observed that the four-jointed box 1 (FJX1) was overexpressed when compared to normal nasopharynx tissue [24]. Although the precise function of FJX1 in human remains unclear, studies carried out using model systems of different species suggests that FJX1 may play key roles in cancer related pathways including Fat and Dachsous [25], Hippo-YAP [26], Notch [27–28] and JAK/STAT signaling [29], pointing to the possibility of the involvement of FJX1 in human carcinogenesis. In support, FJX1 protein has now been reported to be overexpressed in various tumors [30–32] and also correlating in poor survival in colorectal cancer patients [33]. Notably, the human FJX1 chromosomal region, is reported to be amplified and overexpressed in oral cancer tissues and cell lines [34–35], suggesting that the protein may be a viable tumor antigen for head and neck cancers including oral cancer and NPC. In this regard for NPC, we have verified in an independent tissue sample set of NPC tumors and corresponding normal that FJX1 was broadly overexpressed at both the mRNA and protein level [36]. Furthermore, we also demonstrated that ectopic overexpression of FJX1 in NPC cells increased proliferation, anchorage-independent growth and invasive capacity of the cells, suggesting the overexpression of this protein may be contributing to NPC pathogenesis [36]. Notwithstanding, elevated levels of FJX1 in NPCs and other cancers, and combined with the minimal expression in normal human vital organs suggested that this tumor antigen may have value for developing a cancer vaccine that offered benefit as well as fewer side effects to NPC patients.

In this study, we designed 6 MHC class I (HLA-A2 restricted) and 2 MHC class II (HLA-DR restricted) FJX1-derived sequence specific peptides (9–20 amino acids) and evaluated *in vitro*, their potential to stimulate NPC patient’s immune response towards target cancer cell lines overexpressing FJX1. Here, we demonstrate that all peptides were immunogenic and are able to induce anti-tumor immune responses against FJX1-expressing head and neck cancer cells, suggesting that FJX1 specific peptides could be efficacious and a viable therapy for the treatment of head and neck cancers including NPC.

## Materials and Methods

### Patient selection

HLA-A2 is one of the common allele among the Chinese and the Malay sub-population in Malaysia and is also observed in the majority of NPC patients from this region [37–39]. Six HLA-A2 healthy donors and 11 NPC patients from 3 referral hospitals (Kuala Lumpur Hospital, Serdang Hospital and Sime Darby Medical Centre) in Malaysia were recruited for this study. The purpose of the study was explained to both patient cohorts and healthy donors; written informed consent was obtained from all individuals. This study obtained ethical approval from the Medical Research and Ethics Committee, Ministry of Health, Malaysia (NMRR-09-944-4848) and Sime Darby Medical Centre Ethics Committee (EC201109.5). Demographic information of both NPC patients and healthy donors are shown in Table 1. Of the 11 NPC patients tested, 5 were confirmed as HLA-A2 positive, 5 were HLA-A2 negative and for 1 patient (HKL-09), the HLA type could not be determined and consequently omitted from the study. All healthy donors were HLA-A2. Four patient samples and 4 donor samples which were HLA-A2 positive were used for *ex vivo* immunogenicity and post-expansion ELISPOT to determine the efficacy of each peptide. Independent HLA-A2 patient samples and 5 donor samples (3 overlapped with previously mentioned assays) were used to determine the optimum effector: target ratio (E:T). Three HLA-A2 healthy donors and 6 NPC patient samples (4 non-HLA-A2 and 2 HLA-A2 patients) were subjected to proliferation assay to evaluate the efficacy of MHC class II (HLA-DR restricted) long peptides (Table 1).

**Table 1:** Summary on donors/ patients samples used in each assay.

Donor/ Patient Code	Clinical info	Age	Gender	Ethnicity	HLA-type	Immunogenicity	Efficacy	E:T ratio	Proliferation
D-01	Healthy	30	Female	Chinese	A2	√	√	√	
D-02	Healthy	26	Female	Chinese	A2	√	√		√
D-03	Healthy	28	Female	Chinese	A2	√	√	√	√
D-05	Healthy	27	Female	Chinese	A2	√	√	√	√
D-07	Healthy	37	Female	Chinese	A2			√	
D-08	Healthy	39	Female	Chinese	A2			√	
HKL-02^a^	Disease	N/A	N/A	N/A	A2	√	√		
HKL-03	Disease	50	Male	Chinese	A2	√	√		
HKL-04	Disease	74	Male	Chinese	non A2				√
HKL-05	Disease	54	Female	Malay	non A2				√
HKL-06	Disease	56	Male	Malay	non A2				√
HKL-07	Disease	40	Male	Chinese	A2	√	√		
HKL-09^b^	Disease	54	Male	Chinese	Not determined	-	-	-	-
SDMC-01	Disease	35	Female	Chinese	Non A2				√
SDMC-02	Disease	35	Male	Chinese	A2	√	√		√
SDMC-03	Disease	41	Female	Chinese	A2			√	
SDH-01	Disease	48	Female	Chinese	Non A2				√

^a^ N/A: Records not available

^b^ HLA subtype not determined and thus excluded from the study.

### Quantitative PCR

Total RNA from 8 biopsies, 10 NPC cell lines (CNE1, HK1, HONE1, HONE-Akata, SUNE1, TW01, TW04, C666.1, C666.1-A2/control, C666.1-A2/FJX1) and ORL-195 oral cancer cell line were extracted using the Nucleospin RNA II Purification Kit (Macherey-Nagel, Germany). These RNA was used to synthesis cDNA using oligo (dT) primer and Superscript II (Invitrogen, USA). Quantitative PCR was performed with standard SYBR Green protocol using ABI Prism 7500 Sequence Detection System (Applied Biosystems, Germany). GAPDH was amplified and served as an internal control. The primers for human FJX1 to detect mRNA levels were: (forward) 5’-CCCGCAAAGGTGTCTAAAAACT-3’ and (reverse) 5’-GTGCTGGCACAGTAAAGAATCCT-3’. A minimum of 2-fold increase in the relative expression was considered overexpressed when compared to expression levels of normal nasopharynx tissues.

### Peptide design/ synthesis

Six MHC class I (HLA-A2 restricted) and 2 MHC class II (HLA-DR restricted) FJX1-derived sequence specific peptides (9–20 amino acids) were predicted using the BIMAS HLA Peptide Binding Predictions software [http://www-bimas.cit.nih.gov/molbio/hla_bind/] [40] and SYFPEITHI Epitope Prediction program [http://www.syfpeithi.de/] [41]. Peptides with high binding score predicted by either or both BIMAS and SYFPEITHI were shortlisted. All peptides including the HLA-A2 restricted positive control FluM peptide (GILGFVFTL), were synthesized by JPT Peptide Technologies GmbH, Germany, using Good Clinical Laboratory Practice (GCLP). The purity of peptides was determined using high performance liquid chromatography (HPLC) and all were above 80%.

### Cell lines

Two HLA-A2 expressing head and neck cancer cell lines (NPC: C666.1-A2; oral cancer: ORL-195) were used in this study. C666.1 represents an undifferentiated NPC cell line established from a tumor biopsy from a patient of Southern Chinese origin [42] and C666.1-A2 cells (parental C666.1 cells overexpressing the human HLA-A2) was kindly provided by Dr. Ricardo Dolcetti (Centro di Riferimento Oncologico, Italy). The ORL-195, an oral cancer cell line derived from a tumor from the buccal mucosa of a 61 year old patient was established in our laboratory and described by Hamid et al. [43]. Both lines were cultured in their recommended media (C666.1-A2: RPMI-1640; ORL-195; DMEM/F12, both from Gibco, NZ) and supplemented with 2 mM L-glutamine (Sigma-Aldrich, USA) and 10% fetal bovine serum (FBS) (Gibco, NZ). ORL-195 was authenticated by STR profiling using The AmpFlSTR Identifiler PCR Amplification Kit (Applied Biosystems, USA) and compared to patient’s tissue, while C666.1-A2 was authenticated using the GenePrint10 System (Promega, USA) and compared to the STR profile of the original stock when the line was initially established.

### Cytokines

Human recombinant interleukin (IL)-2, IL-4, IL-7, IL-12 and granulocyte macrophage colony-stimulating factor (GM-CSF) were purchased from R&D System (USA).

### FJX1-expressing cancer cell lines

Pivotal to these experiments is the requirement to screen for cancer cell lines with the correct HLA-A2 genotype with high FJX1 levels for use as our target cells in our experiment. Although the CNE2, a NPC cell line is reported to be HLA-A2 positive [44], subsequent studies have revealed that several NPC cell lines, including CNE2 are likely contaminated with the HeLa/ HPV genome. Thus, usage of these compromised cell lines could lead to misleading results and prompted us to adopt an alternative strategy for NPC [45–46]. As C666.1 cells remain free of HeLa/HPV contamination and as mentioned above, with the availability of C666.1-A2 cells, we opted to use this NPC model for our experiments. However, while engineered to express the correct HLA sub-type, endogenous levels of FJX1 were found to be low. To address this limitation and to keep to a clean model system, we ectopically overexpressed FJX1 in these cells using the pLenti6.3/V5-TOPO system (Invitrogen, USA). Cells were cultured with the presence of blasticidin (5 μg/ml) to ensure the selection of only the FJX1 overexpressing clones and the levels of FJX1 were checked prior to experiments to ensure that these remained elevated. Expression of FJX1 was confirmed and checked between experiments, using quantitative PCR (as above) and western blotting using a polyclonal rabbit anti-FJX1 (1: 2000; Aviva System Biology, USA; Cat#: ARP47013_P050). Anti-pan-actin was used as a loading control (1:1000; Cell Signaling, USA; Cat#: MAB1501). Secondary antibodies used were goat anti-rabbit and goat anti-mouse (1: 10000; Cell Signaling, USA), respectively. Overexpressing FJX1 (C666.1-A2/FJX1) and control (C666.1-A2/control) cells were used for subsequent studies. Western blotting technique was used as previously described [47].

As FJX1 has been reported to be overexpressed in oral cancer [34–35], we sought to assess the relative expression levels of this molecule in our oral cancer cell line panel previously established in our laboratory. Here, ORL195 was found to be of HLA-A2 genotype and with endogenously elevated levels of FJX1 and therefore chosen as an oral cancer target line for the post-expansion ELISPOT study as described below.

### Preparation and HLA-typing of patients’ peripheral blood mononuclear cells (PBMC)

Thirty-five milliliters of blood from each of healthy control and NPC patient cohorts, was collected in CPT Vacutainer tubes (Becton Dickinson, USA) and PBMCs were isolated as per manufacturer’s instruction. HLA typing was conducted using the phycoerythrin (PE) tagged mouse anti-human HLA-A2 antibody (clone BB7.2; BD Pharmingen, USA). PE tagged mouse anti-human HLA IgG2b κ-isotype (BD Pharmingen, USA) and mouse anti-human HLA-ABC (clone DX17; BD Pharmingen, USA), were used as negative and positive controls, respectively.

### Peptide binding assay and *ex vivo* ELISPOT assay

T2 cell binding assay was used to evaluate the affinity of the synthesized peptides to HLA-A2 molecules [48]. T2 cells grown in cell suspension essentially lack TAP (transporter associated with antigen processing) protein expression [49]. Therefore, these cells are unable to express stable HLA-A2 molecules on their cell surface and are only able be stabilized by binding to high affinity peptides/antigens. Thus, in our assay, T2 cells were initially incubated with sequence specific peptides for 18–24 hours and after collected by centrifugation (3000 rpm for 10 min). The pelleted cells underwent washing once with phosphate buffered saline (PBS) and resuspending in ~200 μl of PBS, the cell suspension was incubated with 5 μl of phycoerythrin (PE) tagged mouse anti-human HLA-A2 antibody for 45 minutes at 4°C. After incubation, cells were washed once again with 500 μl PBS, resuspended in 400 μl PBS and analyzed using flow cytometry. T2 cells incubated with HLA-A2 restricted FluM peptide (GILGFVFTL) were used as a positive control, while T2 cells in the absence of peptides to stabilize surface HLA-A2 molecules expression were used as negative control. The binding affinity was evaluated by comparing the mean fluorescence intensity (MFI) of HLA-A2 expression in the presence of peptides to the MFI in the negative control.

The *ex vivo* ELISPOT assay was used to evaluate the presence of inherent peptide specific T-cells. Due to the sensitivity and specificity of ELISPOT to enumerate the frequencies of cytokine secreting cells at single cell level, this approach is frequently used in clinical trials for immune monitoring of patients [50–51]. The *ex vivo* ELISPOT was conducted according to the manufacturer’s instructions (Mabtech AB, Sweden) with slight modifications. PBMCs were incubated with 50 ng/ml of IL-7 and 10 ng/ml of IL-12 at 37°C, for 2 hours. After incubation, PBMCs were washed, resuspended in RPMI-1640 supplemented with 2 mM L-glutamine, 5% human AB serum (Gemini Bio-Products, USA) and incubated with 50 μg/ml of peptides in a 96-well U-bottom plate for 18–24 hours. Following incubation, PBMCs were added to IFNγ/granzyme B coated ELISPOT plate and further incubated for 2 hours at 37°C. This was followed by the addition of IFNγ/granzyme B detection antibody and incubation for an additional 2 hours and after, streptavidin tagged secondary antibody and incubation for 45 minutes, both at 37°C. Both antibodies were diluted at 1:500 in PBS containing 0.5% human AB serum (Gemini Bio-Products, USA). BCIP/NBT-plus substrate was added to visualize spots formed by cytokine-secreting T-cells. The detected spots were then quantitated using CTL ELISPOT Analyzer (Cellular Technology Limited, Ohio, USA) and analyzed using the ImmunoSpot Professional Software (Cellular Technology Limited, Ohio, USA). We have previously compared the number of spots obtained in the ELISPOT assay using the irrelevant peptide (HIV-A2) compared to no peptide control using PBMC from 2 independent subjects and similar results (number of spots) were observed in the *ex vivo* ELISPOT assay. Therefore, we chose to use no peptide control in all subsequent experiments. Peptide-specific CTLs were calculated by subtracting spots formed in the negative control wells with no exposure to peptide.

### Dendritic cells (DCs) and cytotoxic T-lymphocytes (CTLs) isolation and cultures

Dendritic cells and T-cells were isolated from patient’s PBMC as described previously [52] with slight modifications. Briefly, PBMCs were resuspended in macrophage-serum free media (M-SFM) (Gibco, NZ), seeded in a 6-well plate and incubated for 1 hour at 37°C, After incubation, non-adherent cells from PBMCs were removed and resuspended in RPMI-1640 supplemented with 2 mM L-glutamine, 5% human AB serum (Gemini Bio-Products, USA) and 10 ng/ml IL-7. Approximately 2–5 x10^5^ cells per well were seeded into round-bottom 96-well plates. Cells were than incubated for 2–5 days at 37°C for T-cell expansion. The adherent cells from the PBMC population in the 6-well plate were cultured at 37°C for 2–5 days in M-SFM in the presence of 100 ng/ml GM-CSF and 25ng/ml IL-4 to induce differentiation to dendritic cells (DCs). Differentiated DCs were collected from the culture dish by dislodging the cells with media and then aliquoted into 1.5ml centrifuge tube, incubated with 50 μg/ml peptides for 2 hours at 37°C before applying to the matured T-cells derived from nonadherent PBMCs. After co-culture for 18–24 hours, 10 ng/ml of IL-2 was added to the DCs/T-cell mix and incubated for an additional 24 hours. After incubation, culture media containing IL-2 was replaced with fresh media and DC-pulsed T cells were cytokine starved for 3–5 days to allow the activation of cytotoxic T-lymphocytes (CTLs). CTLs were then co-incubated with target cells (ORL195, C666.1-A2 control and C666.1-A2/FJX1) in the post-expansion ELISPOT assay (described below) to determine the potential killing ability of CTLs against FJX1-expressing target cells.

### Post-expansion ELISPOT assay

CTLs were mixed with ORL195 cells at an effector to target ratio of 1: 1, and this cell mix was supplemented with 20 ng/ ml of IL-7 and IL-2 and incubated at 37°C for 18–24 hours. After incubation, cells were resuspended in 200 μl of RPMI-1640 and then applied 100 μl/well to the IFNγ/granzyme B ELISPOT plate and further incubated for 2 hours at 37°C. ELISPOT assay was than performed as described above.

Peptide specificity was confirmed by carrying out post-expansion ELISPOT assay using FJX1 expressing NPC cells (C666.1-A2/FJX1) and C666.1-A2/control cells at effector to target ratio of 1: 1, 5: 1, 10: 1 and 20:1. The optimal E:T ratio was determined when increased T-cells cytotoxic potential (secretion of IFN-γ and granzyme B) was detected when FJX1 expressing NPC cells (C666.1-A2/FJX1) was used compared to C666.1-A2/control cells.

### T-cells proliferation assay

PBMCs from healthy donors and NPC patients were seeded in a 96-well flat bottom culture plate at the density of 2 x 10^5^ cells/ well. Fifty μg/ml of MHC class II peptides (Pep-07 and Pep-08) or 0.5μg/ml of the phytohaemagglutinin (PHA) were added to respective wells and incubated for 3–5 days at 37°C. The proliferation of T-cells was examined using Cell Proliferation BrdU ELISA Colorimetric Kit (Roche Diagnostics GmbH, Germany) as per the manufacturer’s instructions. PBMCs stimulated by PHA were used as a positive control while the wells seeded with PBMCs cultured in the absence of peptide served as negative controls. T-cell proliferation induced by MHC class II peptides was determined by measuring the absorbance at 450 nm after subtracting the negative control wells.

### Statistical analysis

Statistical analyses were performed using the SPSS statistical analysis package (Version 16.0, SPSS Inc., Chicago, USA). Unpaired Student T-test was used to compare differences of T-cells enumerated between groups. *P*-value < 0.050 was considered to be statistically significant.

## Results

### FJX1 is a potential tumor antigen overexpressed in NPC

**Fig 1:**
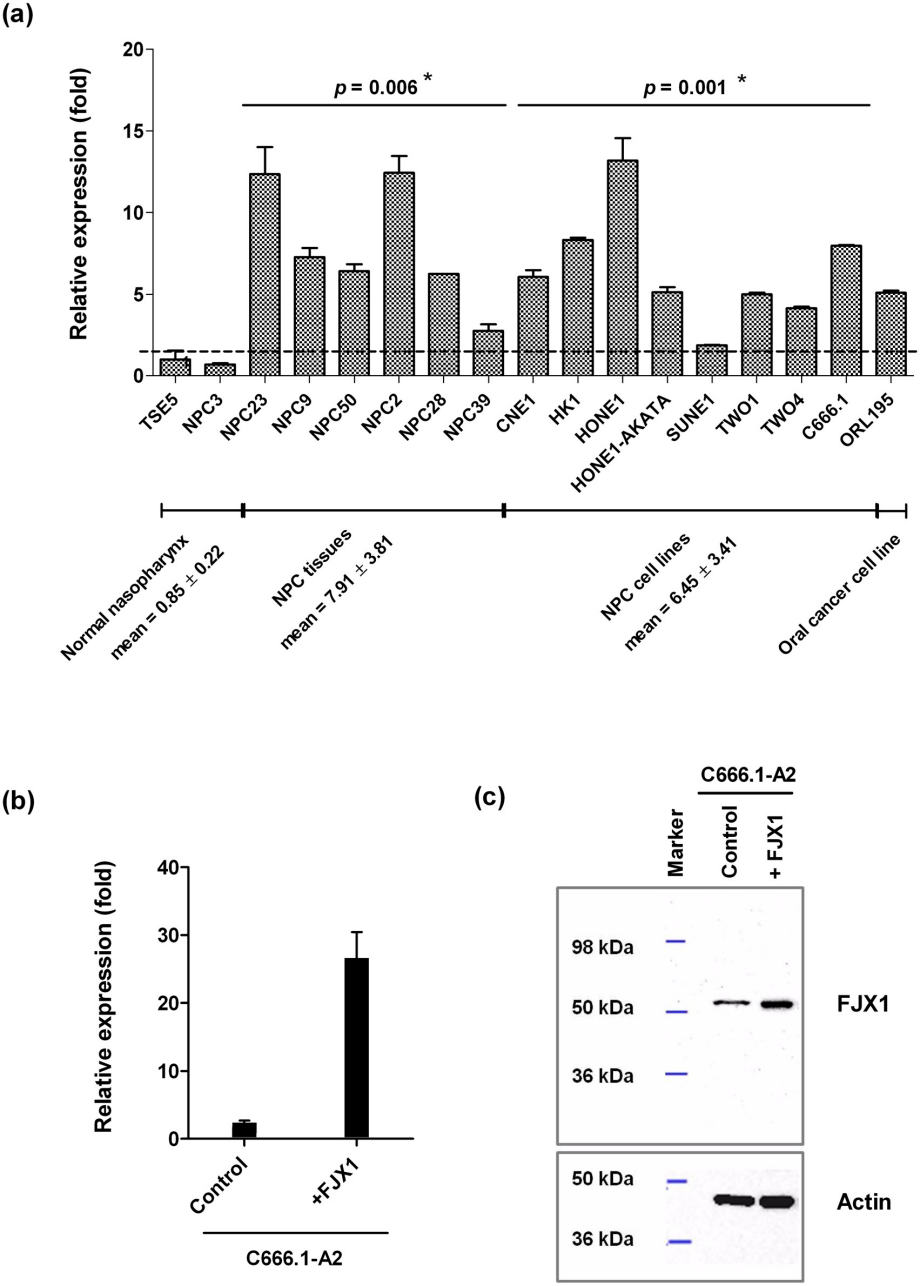
Overexpression of FJX1 in biopsies and cell lines. (a) Quantitative PCR on NPC cell lines, oral cancer cell line and biopsies from NPC patients showed increased levels of FJX1 mRNA compared to the normal nasopharynx tissue (TSE5 and NPC3). FJX1 is considered to be overexpressed when the relative expression ≥ 2 fold is observed when compared to normal. FJX1 overexpression in C666.1-A2 and corresponding control counterpart cell lines was confirmed at both (b) mRNA and (c) protein level.

Confirming the observations from our microarray study [24], we demonstrated that the mRNA levels of FJX1 is ~7 fold elevated in a small subset of untreated NPC tissues (n = 6; *p* = 0.006) and NPC cell lines (n = 9; *p* = 0.001) when compared to 2 normal nasopharynx tissues (TSE5 and NPC3) as shown in Fig 1a. C666.1 and ORL195 cells showed elevated levels of 8 and 5 fold, respectively. Expression levels of FJX1 were also assessed in C666.1-A2/FJX1 and C666.1-A2/control cells. As shown in Fig 1b, mRNA levels in C666.1-A2/FJX1 cells were highly elevated (~26 fold) compared to C666.1-A2/control. Notably, this increment in mRNA was also reflected at the protein level (Fig 1c). Pan-actin was used to control loading, FJX1-specific band at ~50 kDa was seen to be elevated compared to the control counterpart. On-going studies in our laboratory have shown that large fraction of our oral cancer cell line panel including ORL195 express high levels of FJX1 protein. With this information, ORL195 together with the C666.1-A2/FJX1 and C666.1-A2/control cells were used for subsequent experiments.

### Identification of FJX-1 peptides with high binding affinity to HLA-A2

**Fig 2:**
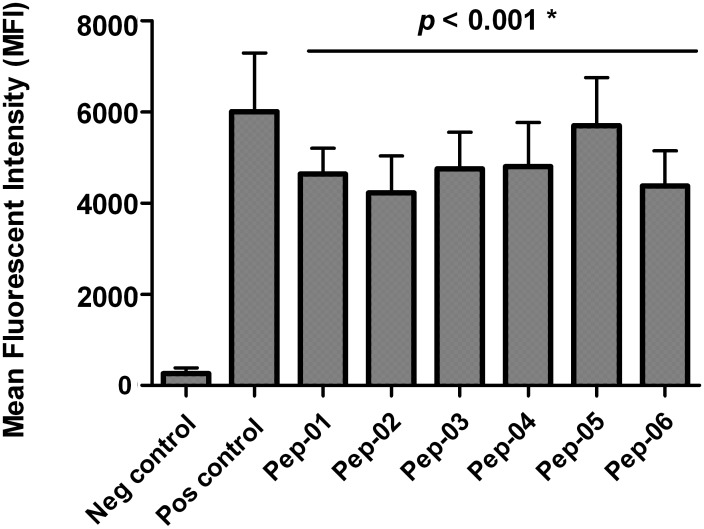
HLA-A2 peptides demonstrated high binding affinity towards MHC class I molecule. All 6 HLA-A2-restricted peptides demonstrate higher binding affinities towards HLA-A2 molecules suggesting stabilization of HLA-A2 molecule expression on T2 cell surface compared to the negative control (*p* < 0.001). Binding affinity was evaluated by comparing the mean fluorescence intensity (MFI) of HLA-A2 expression in the presence of peptides compared to the MFI in the absence of peptide. HLA-A2 restricted FluM-derived peptide was used as a positive control and samples with the absence of peptide were used as negative control.

Six HLA-A2 specific peptides (Pep-01 to Pep-06; 9–11 amino acids) and 2 MHC class II (HLA-DR restricted) peptides (Pep-07 and Pep-08; 18–20 amino acids) identified by BIMAS and SYFPEITHI epitope prediction software were synthesized (Table 2). Pep-08 was designed as a multi-epitope peptide to encompass 2 HLA-A2 binding motifs constituting Pep-05 and Pep-06. The six HLA-A2 peptides were tested using the T2 binding assay and compared to the negative control where cells were not exposed to the peptides. Mean fluorescent intensity (MFI) that was determined essentially reflects the amount of stabilized HLA-A2 molecules on T2 cells. As shown in Fig 2, all 6 HLA-A2-restricted peptides bound and stabilized the expression of MHC class I molecules on the surface of T2 cells when compared to negative control (*p* < 0.001). Broadly, the binding affinities of all 6 peptides were similar and comparable to the binding affinity of the positive control (FluM peptide). Flow cytometry analysis of stabilized MHC class I expression in T2 cells is shown in S1 Fig. Overall the data shows that these FJX1-derived peptides have good binding affinity and are able to stabilize the HLA-A2 expression on TAP-deficient T2 cells.

**Table 2:** FJX1 sequence-specific peptides.

Peptide name	MHC Class	Location	Peptide Sequence	Length
Pep-01	I	aa 15 –aa 25	WLLALGSLLAL	11 aa
Pep-02	I	aa 28 –aa 36	GLLPPRTEL	9 aa
Pep-03	I	aa 192 –aa 200	ALSYYLARL	9 aa
Pep-04	I	aa 304 –aa 312	RLVSNLFSL	9 aa
Pep-05	I	aa 404 –aa 412	ALADPHAQL	9 aa
Pep-06	I	aa 412 –aa 420	LLQRRLDFL	9 aa
Pep-07	II	aa 74 –aa 91	GGSLKTFRALLTLAAGAD	18 aa
Pep-08	II	aa 401 –aa 420	ELAALADPHAQLLQRRLDFL	20 aa

Six HLA-A2 restricted peptides and 2 MHC class II peptides were predicted using epitope prediction software and synthesized using Good Clinical Laboratory Practice (GCLP).

### *Ex vivo* ELISPOT demonstrate the presence of FJX1-specific T-cells in the PBMCs of healthy donors and NPC patients

**Fig 3:**
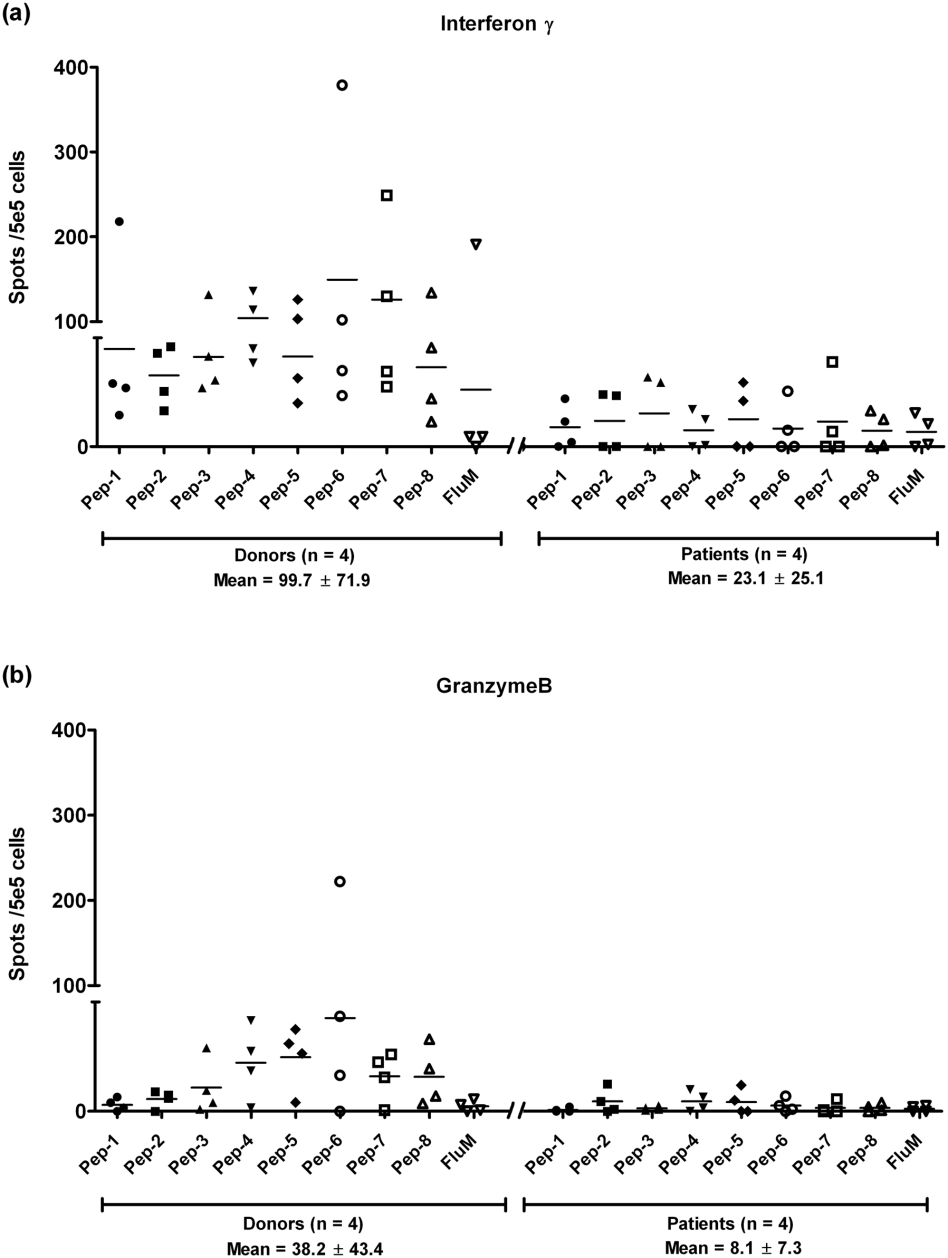
FJX1 sequence specific peptides are immunogenic. (a) *Ex vivo* ELISPOT demonstrated higher secretion of IFN-γ in healthy donors (n = 4) when compared to patients (n = 4; *p* = 0.002). (b) *Ex vivo* ELISPOT demonstrated higher secretion of granzyme B in donors (n = 4) when compared to patients (n = 4; *p* = 0.003).

After demonstrating that the FJX1 short peptides have high binding affinity towards HLA-A2 molecules on T2 cells, we sought to assess if the predicted HLA-A2 epitopes could be recognized by PBMCs of healthy donors and NPC patients’. As shown in Fig 3a and 3b, all peptides to a degree, were able to induce the secretion of IFN-γ and granzyme B in both donors (n = 4) and patients (n = 4) indicating the presence of peptide-specific T-cells in the PBMC from both sample cohorts. However, differences of ~4-fold more FJX1-specific IFNγ-secreting (healthy donors: NPC patients = 99.7 ± 71.9: 23.1 ± 25.1, *p* = 0.002) and granzyme B-secreting T-cells (healthy donors: NPC patients = 38.2 ± 43.4: 8.1 ± 7.3, *p* = 0.003) in healthy donors when compared to NPC patients were observed. Overall, the data suggests that all the peptides are immunogenic and able to be recognized by inherent T-cells and react by secreting cytokines. Using an independent set of PBMCs (Table A and B in S1 File), we demonstrated no significant differences between spots generated from the wells using HIV-A2 peptides and the wells with absence of peptides (Table C in S1 File). Hence, spots from negative controls wells with absence of peptide were considered as background and were subtracted from the data.

### FJX1 peptides induce antigen-specific cytotoxic cytokines secretion activity *in vitro*

**Fig 4:**
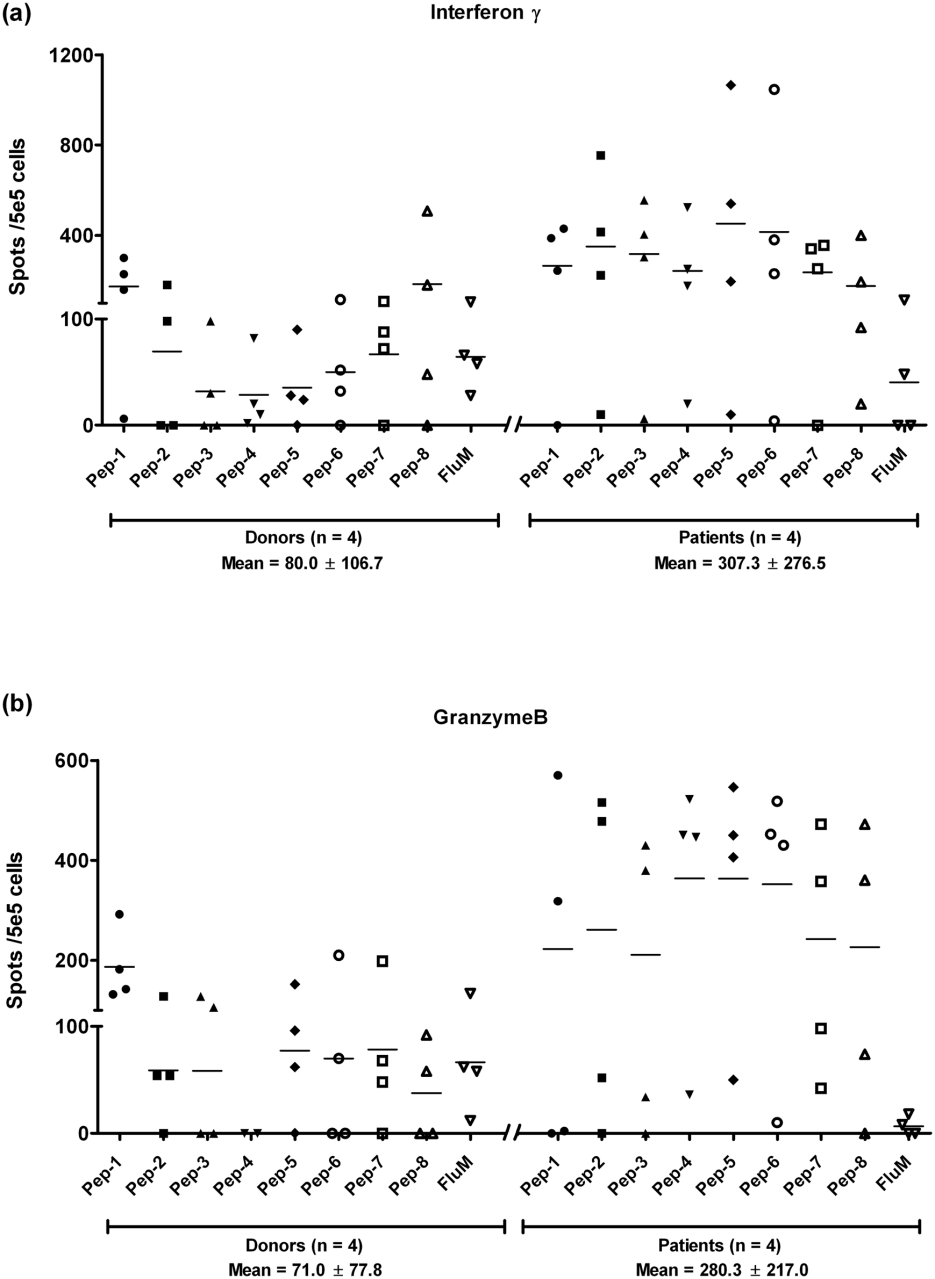
FJX1 peptides stimulate higher cytokine secretion in NPC patients PBMCs when compared to donors equivalent after peptide pulsation. (a) Augmentation of IFN-γ secreting CD8+ T-cells was significant higher in NPC patients (n = 4) when compared to healthy donors (n = 4) after peptide stimulation (*p* = 0.003). (b) Augmentation of granzyme B secreting CD8+ T cells was significant higher in NPC patients (n = 4) as compared to healthy donors (n = 4) after peptide stimulation (*p* < 0.001). Spot numbers was calculated by subtracting spots in the negative control wells with the absence of peptides.

Having shown that FJX1 peptides can be recognized by inherent T-cells from healthy donors and NPC patients, we next sought to assess if the peptides are able to enhance T-cell immune response and target recognition after presented by antigen presenting DCs. To determine the cytotoxic potential of stimulated T-cell cultures after co-incubation with DCs presenting the peptides, stimulated T-cells cultures were then co-incubated with one of the target cells lines, ORL195. T-cells population that recognized the target cell line and secretes IFN-γ/ granzyme B were measured using the ELISPOT assay. Broadly, all peptides were capable of stimulating T-cells from both healthy donors (n = 4) and patients (n = 4), where the mean cytokine-secreting CTLs was significantly higher in T-cells from NPC patients compared to healthy donors for both IFN-γ (healthy donors: NPC patients = 80.0 ± 106.7: 307.3 ± 276.5; *p* = 0.003) and granzyme B (healthy donors: NPC patients = 71.0 ± 77.8: 280.3 ± 217.0; *p* < 0.001) (Fig 4a and 4b). Another set of ELISPOT assays using C666.1-A2/FJX1 as target cells were set up and as seen we also observed an enhanced immune response in these 5 healthy donors (3 overlapped) and 3 NPC patients, suggesting that training of T-cells with FJX1-derived peptides can likely stimulate T-cell responses against cells expressing FJX1 regardless of the cell type (Figures A and B in S2 File). Broadly, FJX-1 specific might be beneficial for cancers expressing FJX-1 regardless of the cancer origin.

**Fig 5:**
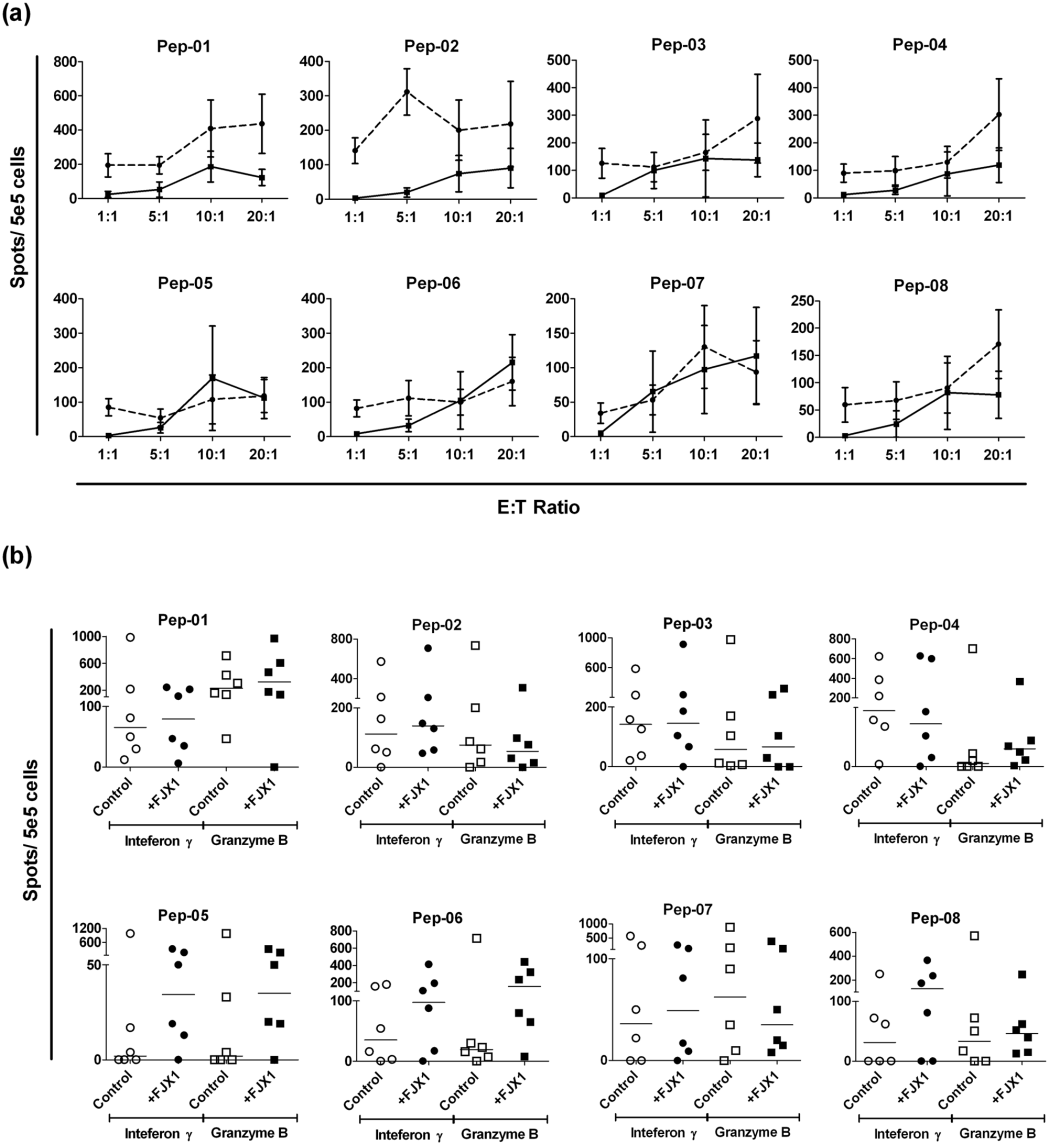
FJX1-derived peptides are able to activate T-cells to recognize cancer cells using an optimal E:T (effector: target) ratio. (a) Optimal cytotoxic potential of post-expansion T-cells was observed at E: T ratio of 20: 1, where most of the peptides were able to induce subjects’ T-cells secreting high level of IFNγ and granzyme B. Dotted lines represent granzyme B secretion and the solid lines represent IFNγ secretion. (b) Pep-01, Pep-03, Pep-05, Pep-06 and Pep-08 demonstrated a trend of specificity where the peptides were able to activate T-cells to recognize FJX1 overexpressing cancer cells (C666.1-A2/FJX1).

To determine the most efficacious ratio between stimulated T-cells (effectors) against cancer cells (targets), we co-incubated our target cell line (C666.1-A2/ FJX1) with different effector: target ratio. For IFN-γ secretion, 4 of the 8 peptides (Pep-01, Pep-03, Pep-05, Pep-08) showed the highest secretion at E: T ratio of 10: 1, while remaining (Pep-02, Pep-04, Pep-06, Pep-07) gave the highest secretion at 20: 1. On the other hand, 1 of 8 (Pep-07) showed high granzyme B secretion at 10: 1 while 6 of 8 peptides (Pep-01, Pep-03, Pep-04, Pep-05, Pep-06 and Pep-08) gave the highest secretion at 20: 1 (Fig 5a). This suggest that E:T ratio of 20:1 was determined to be the most efficacious ratio in eliciting FJX1-specific immune responses. We next tested this E:T ratio of 20:1 with the same peptides set as indicated above and C666.1-A2/FJX1 as the target line. Notably, Pep-01, Pep-03, Pep-05, Pep-06 and Pep-08 were able to provoke T-cells into secreting higher levels of IFNγ and granzyme B when compared with C666.1-A2/control cells, suggests that these peptides are specific towards FJX1 overexpressing cells (Fig 5b).

### MHC class II peptides induced CD4+ T_H_ cell proliferation

**Fig 6:**
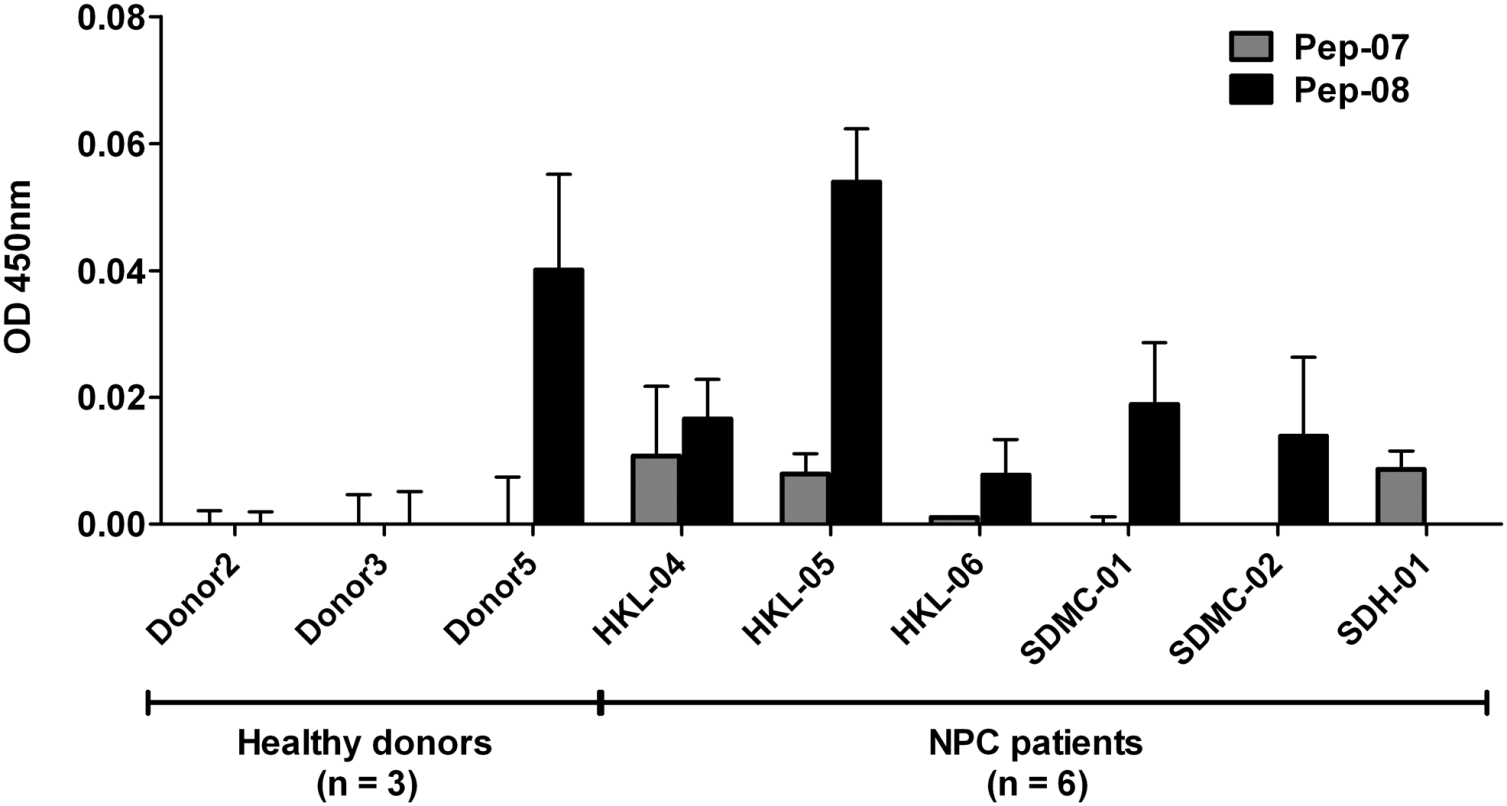
MHC class II peptides induced CD4 T-cell proliferation. Pep-08 was able to induce T-cell proliferation in 6/9 of total samples while Pep-07 was only able to induce T cell growth in only 3/9 total samples tested.

To determine whether the two FJX1 MHC Class II HLA-DR restricted peptides (Pep-07 and Pep-08), can stimulate the expansion of CD4+ T helper (T_H_) cells; we conducted T-cell proliferation assay as described in Materials and Methods. As seen in Fig 6, our results demonstrate that Pep-08 was able to induce T-cell proliferation in 6 of 9 samples tested (healthy donor: 1 of 3; NPC patients: 5 of 6) while on the other hand, Pep-07 was able to induced T-cell proliferation in 3 of 9 NPC patients, suggesting that Pep-08 is likely more potent MHC class II peptide compared to Pep-07 but due to small sample size this difference is not considered significant. A note to mention, although our sample size overall is small, we nevertheless observed that the T-cell proliferative response was more robust in NPC patients compared to healthy donors, where T_H_ cell proliferation was observed in all six patients but only in one out of the three healthy donors.

## Discussion

Many NPC patients in endemic regions are of Chinese origin and predominately HLA-A2 positive [37–39]. Therefore in this study, we chose to design FJX1-specific HLA-A2-restricted peptides and MHC class II HLA-DR restricted long peptides and these was then evaluated as potential cancer vaccine targets in FJX1 overexpressing NPCs.

Cancer vaccines against NPC have largely focused on targeting proteins encoded by the EBV due to their strong correlation with the disease [53–54]. Studies on non-EBV related tumor antigens for developing cancer vaccines still remain elusive. To the best of our knowledge, this is the first study describing the feasibility of using a non-EBV tumor antigen as vaccine target for NPC. In this study, we chose to design and evaluate the efficacy of short-peptides specifically targeted against FJX1, a tumor antigen that we have previously observed to be highly elevated in NPC, but low in normal nasopharyngeal tissue (Figures A and B in S3 File). Of noteworthy, various normal human organs were largely found to express minimal levels of FJX1 (Figure C in S3 File). While the exact function of human FJX1 is currently unclear, recent reports have suggested a possible functional role in cancer pathogenesis [26]. However, the potential for FJX1 as a target for developing cancer vaccines as an alternative therapy for NPC has not been explored.

The premise of cancer vaccines is essentially to harness the patient’s immune system to recognize and eradicate cancer cells. Many studies have shown that peptides consisting of 9–20 amino acids are safe and well tolerated in cancer patients [55–57]. Reported side effects include grade 1/ 2 local skin reaction with redness or swelling at the injection site, while more serious hematologic, cardiovascular, hepatic or renal toxicities have not been observed during the course of vaccination [58]. Overall, grade 3/4 toxicity, is rare and long-term adverse effects have not been reported thus far [59]. Hence, antigen specific peptide vaccines can offer an approach for targeted therapy with fewer side effects for NPC cancer patients.

In the case of NPC patients, conceptually, the T-cells should be able to recognize FJX1-specific peptides more efficiently compared to healthy individuals that have none or minimal exposure to FJX1. However, in this study, we observed a less robust FJX1-specific cytokine-secreting T-cells response in NPC patients when compared to healthy donors prior to any peptide stimulation, suggesting the presence of immunosuppression in this NPC patient cohort. In this context, immunosuppressive activity has been reported in lung, colorectal and EBV-associated cancer patients [60–62], which can be likely due to the presence of immune suppressing T-cells or immune modulating cytokines [63–64]. However, reports of immune-suppression in NPC patients, remain elusive and an area of active investigation. In this regard and based on our observations, we are currently evaluating if a correlation exists between FJX1 peptide-specific immune response and the expression of FJX1 in patients’ tumors, as well as the presence of immunosuppression or immune checkpoint in NPC patients.

Also, in this study, potential cytotoxic activity of CTLs against tumor cells was measured using the ELISPOT assay. ELISPOT is an indirect measurement of cytotoxic potential, but it has been shown to have better sensitivity, particularly in a low effector: target ratio setting and useful when cancer patients have low lymphocyte count and limited PBMCs. This method has also reported to have an excellent correlation (R^2^ = 0.95) with cytotoxicity measured using the ^51^Cr-release assay [50] and was used as an indicator of functional activity of T-cells [65–66]. Although we observed lower number of inherent FJX1-specific T-cells in patients prior to peptide stimulation compared to healthy donors in the *ex vivo* ELISPOT assay, an increased number of FJX1-specific-cytokine secreting CTLs were detected in all patients (n = 4) after stimulation with peptides. This suggests that FJX1-specific CTL repertoire was not completely exhausted and re-sensitization of the immune system with FJX1-derived peptides may be able to reactivate the T-cells that may have been inactivated or tolerated overtime to re-recognize tumor cells [67]. Interestingly, we noted that it was more effective to induce FJX1-specific CTLs in NPC patients compared to healthy donors in the post-expansion ELISPOT assay. CTLs from healthy donors did not expand significantly followed by *in vitro* culture and stimulation with the peptides. Similar observations have been reported by Marincola et al. [68], where melanoma patients were found to be more readily sensitized by MART-1 specific peptides compared to healthy donors, suggesting that cancer patients and healthy individuals immune system get exposed differently to tumor antigens. The observation from our study supports the fact that NPC patients have inherent CD8+T cells that may likely recognize the FJX1 protein as this protein is highly expressed in NPC, but may require re-sensitization to mount a robust response. Since FJX1 is minimally expressed in healthy donors, the expansion of FJX1-specific immune response in this sample cohort is limited. In addition, the response we observed after peptide stimulation prevails over the negative controls where T-cells were grown in the absence of FJX1-derived peptides, suggesting that the immune response observed is peptide specific.

The *ex vivo* ELISPOT was optimized essentially to detect any inherent FJX1-specific T-cells, while the post-expansion ELISPOT was setup to determine the potential of recognizing the killing activity of these T-cells with the presence of target cell line overexpressing FJX1. Since the experimental design for the *ex vivo* ELISPOT differs from the post-expansion ELISPOT, we did not statistically compare the results of prior- and after-peptide stimulation. Based on BIMAS and SYFPEITHI prediction, these HLA-A2 peptides have high binding scores towards HLA-A2 molecules. Some of the peptides could bind weakly towards other HLA types such as the HLA-A24. Therefore, we cannot rule out the possibility of cross reactivity of these peptides with other HLA types. Also, different HLA-A2 subtypes in healthy donors and NPC patients might also influence the binding and activation of T-cells. Since we did not perform high resolution HLA typing for all healthy donors and NPC patients, we cannot exclude the possibilities of variations in HLA-A2 subtypes in influencing the binding and activation of T-cells. Post-expansion ELISPOT assays with the absence and presence of anti-HLA-A2 or anti-MHC class I antibodies shall provide more information regarding the mechanism of peptides eliciting T-cells response and currently under evaluation in our laboratory. In this study, post-expansion ELISPOT on the specificity assay was broadly found to be non-conclusive. These assays were carried out using PBMCs stimulated with peptides and not the pure clones of antigen specific-T-cells. Therefore, we were unable to observe the distinct differences in our specificity assay. The knowledge generated using peptide-specific T-cell clones is likely to provide overall, a more specific dataset. Our aim of this study however, was to screen all eight peptides efficiently. Hence, we chose to screen the efficacy of these peptides using subjects’ T-cells; however, we aim to generate pure T-cell clones for the shortlisted peptides for future evaluation.

In addition to stimulating CTL activity, the exogenous antigen-presenting pathway through MHC class II molecules remains crucial in generating long lasting memory against antigens through CD4+ T-cells [69–70]. Activation of CD4+ T-cells can subsequently provide the necessary signals to activate and expand the primary CD8+ T-cells, as well as to maintain the memory CD8+ T-cells [71–72]. Co-immunization of MHC class I peptides with corresponding peptides carrying MHC class II epitopes for CD4+ T_H_ recognition was also reported to enhance antigen specific immune response [73–74]. Both MHC class II peptides (Pep-07 and Pep-08) used in this study were able to stimulate T-cell proliferation providing the basis to include further assays to evaluate the long-term immune responses induced by MHC class II peptides to study the effect of MHC class II long peptides.

Although with a limited sample number, our results demonstrate that the FJX1-derived peptides are immunogenic and able to induce anti-tumor immune responses in NPC patients against FJX1-expressing tumor cells irrespective of cancer type. Our data also suggests that these peptides could be a potential treatment strategy for NPC patients and might also benefit other cancers overexpressing FJX1.

## Supporting Information

S1 Fig HLA-A2-restricted peptides stabilized the expression of MHC class I molecules on the surface of T2 cells. All 6 HLA-A2 restricted peptides binds to MHC class I molecules and were stained positively using with HLA-A2 antibody tagged with PE in flow cytometry compared to the negative control. HLA-A2 restricted FluM-derived peptide was used as a positive control and samples omitted peptides was used as negative control.
(TIF)

S1 File Cytokine secretion by PBMCs incubated with irrelevant peptide (HIV-A2) is not significantly different from wells with omitted peptide. The *ex vivo* ELISPOT using PBMCs from 2 healthy individuals: HD-09 **(Table A)**, HD-10 **(Table B)**, do not show significant difference when incubated with HIV-A2 peptide and when peptide is omitted. T-test comparing the triplicate wells from irrelevant peptide (HIV-A2) control and no-peptide control (CM) of 2 healthy individuals showed *p* > 0.050 **(Table C)**.
(PDF)

S2 File FJX1-derived peptides are able to stimulate T-cell responses against C666.1-A2/FJX1 target cells. Secretion of IFN-γ **(Figure A)** and granzyme B **(Figure B)** followed by peptide stimulation was observed in 5 healthy donors and 3 NPC patients.
(TIF)

S3 File The expression of FJX1 is high in NPC samples but low in normal nasopharynx and normal organs. Previous microarray results showed the increased level of FJX1 mRNA transcript in NPC biopsies and NPC cell lines compared to normal nasopharynx tissue **(Figure A)**. Representative normal nasopharynx and NPC were stained for FJX1. 18 out of 43 NPC samples (42%) were positively stained with anti-human FJX1 rabbit polyclonal antibody (Aviva Systems Biology, USA) at 1: 500 dilution in PBS, confirming FJX1 was overexpression at protein level in NPCs. Normal nasopharyngeal tissues were consistently stained negative for FJX1 (0/11) **(Figure B)**. Semi-quantitative PCR using Human MTC Panel I & II (Clonetech, USA) showed low and negligible expression of FJX1 in 16 normal human organs compared to the positive control. cDNA from NPC cell line was used as a positive control **(Figure C)**.
(TIF)
