# Supplementary figures and images for: Identification of Four-Jointed Box 1 (FJX1)-Specific Peptides for Immunotherapy of Nasopharyngeal Carcinoma
Source: PLoS One. 2015 Nov 4;10(11):e0130464. doi: 10.1371/journal.pone.0130464 (PMC4633155; doi:10.1371/journal.pone.0130464)

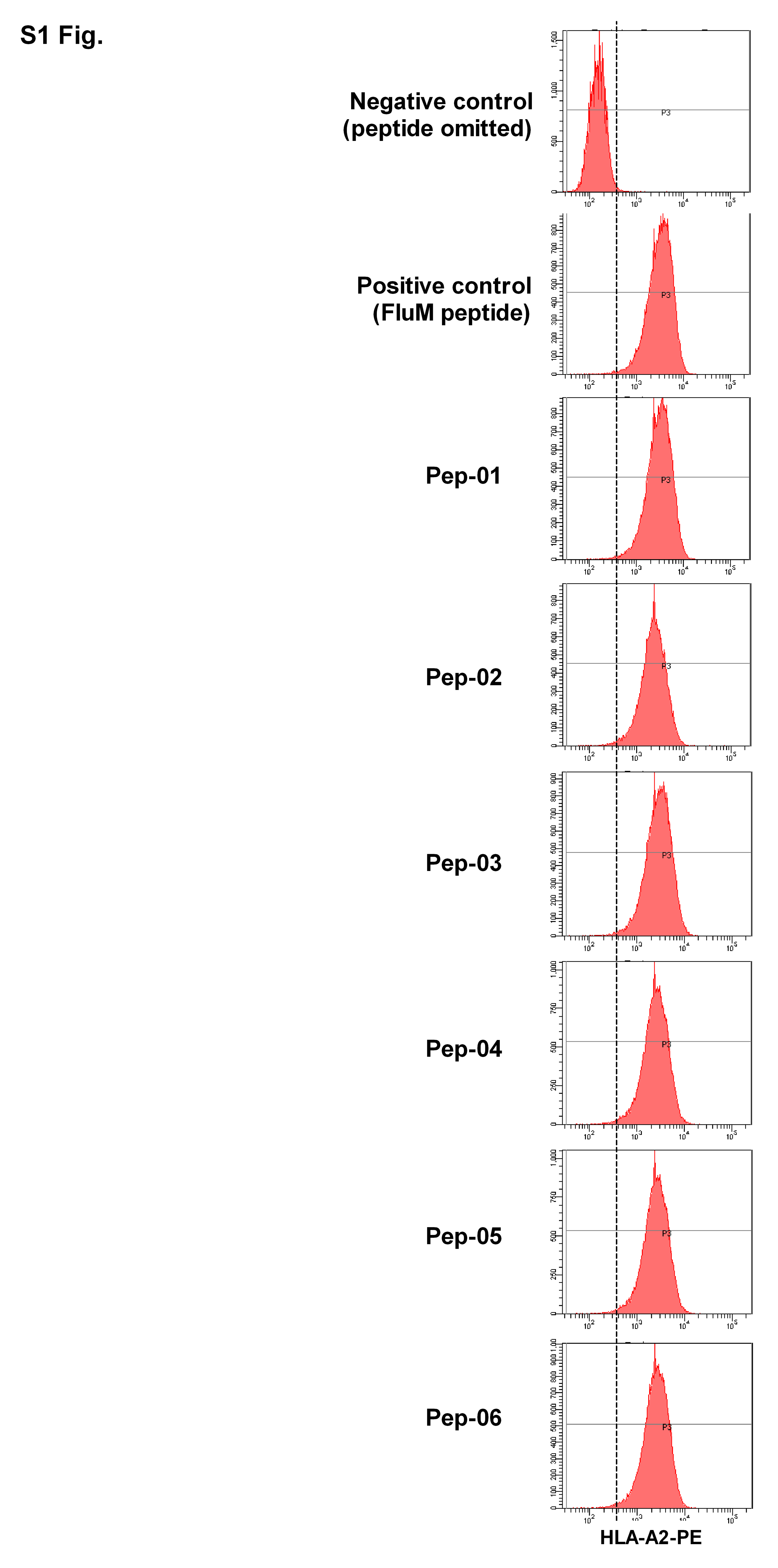

Supplement: S1 Fig — All 6 HLA-A2 restricted peptides binds to MHC class I molecules and were stained positively using with HLA-A2 antibody tagged with PE in flow cytometry compared to the negative control. HLA-A2 restricted FluM-derived peptide was used as a positive control and samples omitted peptides was used as negative control. (TIF) [file pone.0130464.s001.tif]

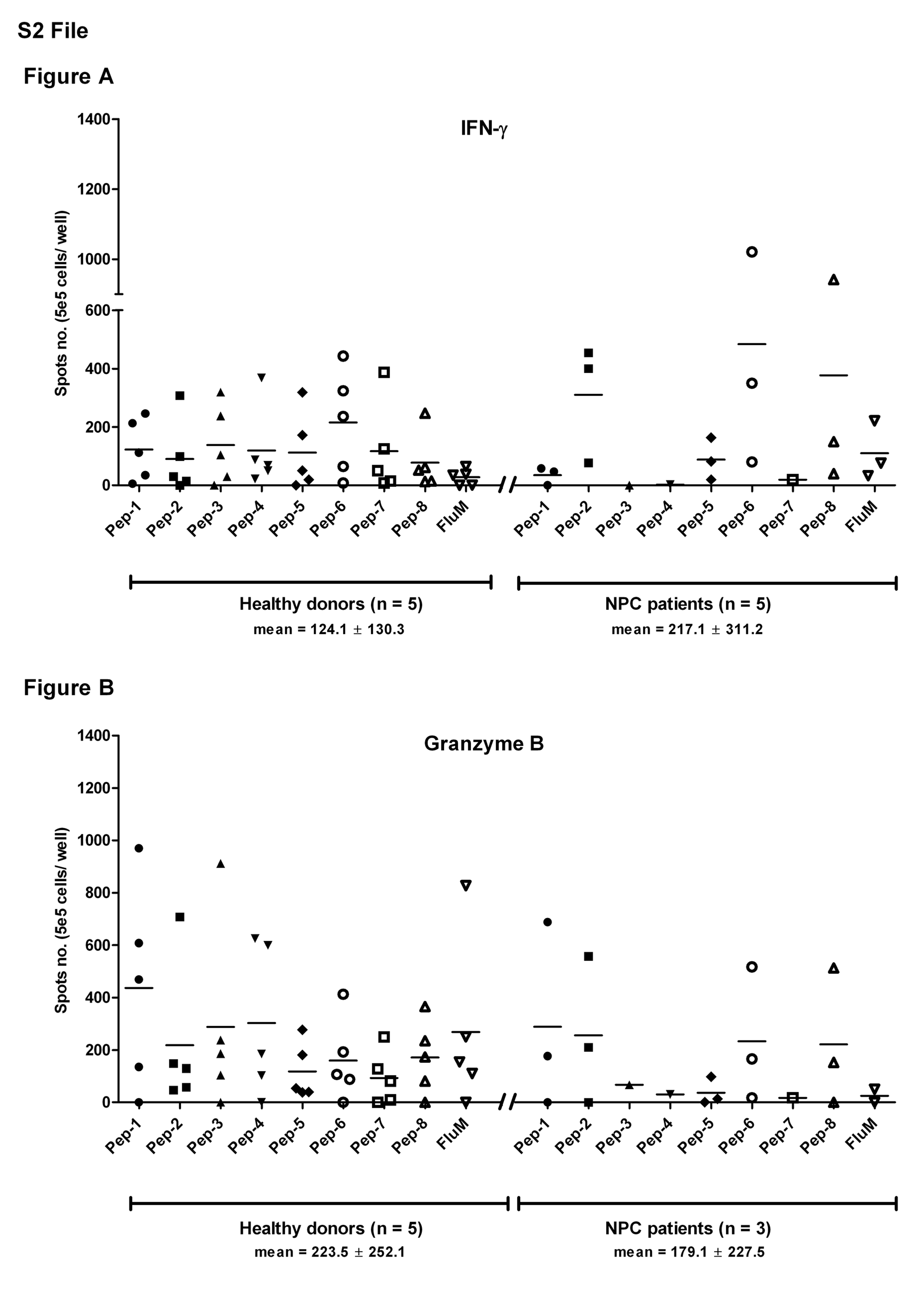

Supplement: S2 File — Secretion of IFN-γ (Figure A) and granzyme B (Figure B) followed by peptide stimulation was observed in 5 healthy donors and 3 NPC patients. (TIF) [file pone.0130464.s003.tif]

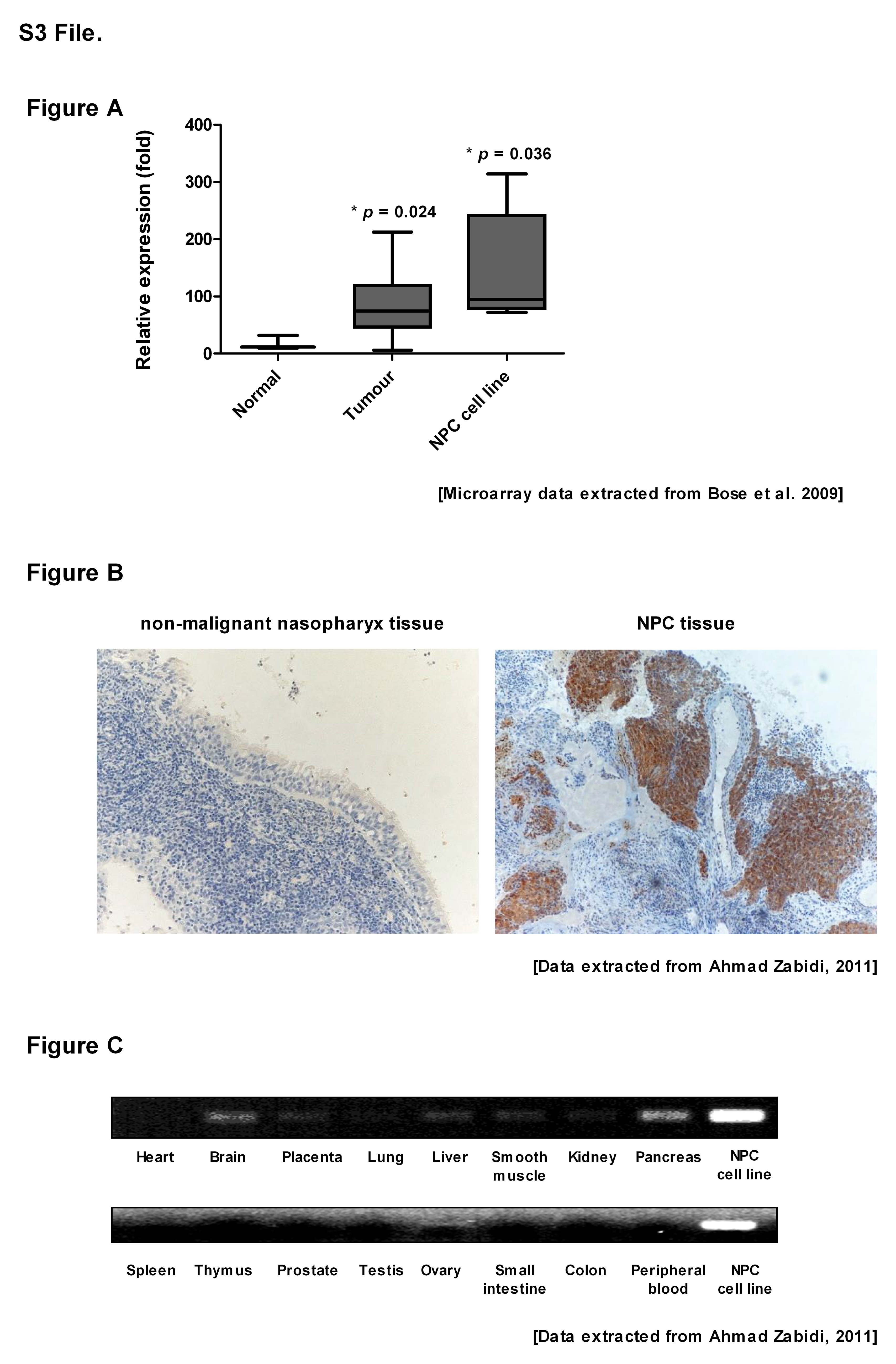

Supplement: S3 File — Previous microarray results showed the increased level of FJX1 mRNA transcript in NPC biopsies and NPC cell lines compared to normal nasopharynx tissue (Figure A). Representative normal nasopharynx and NPC were stained for FJX1. 18 out of 43 NPC samples (42%) were positively stained with anti-human FJX1 rabbit polyclonal antibody (Aviva Systems Biology, USA) at 1: 500 dilution in PBS, confirming FJX1 was overexpression at protein level in NPCs. Normal nasopharyngeal tissues were consistently stained negative for FJX1 (0/11) (Figure B). Semi-quantitative PCR using Human MTC Panel I & II (Clonetech, USA) showed low and negligible expression of FJX1 in 16 normal human organs compared to the positive control. cDNA from NPC cell line was used as a positive control (Figure C). (TIF) [file pone.0130464.s004.tif]
