# Supplementary material for: Identification of Four-Jointed Box 1 (FJX1)-Specific Peptides for Immunotherapy of Nasopharyngeal Carcinoma
Source: PLoS One. 2015 Nov 4;10(11):e0130464. doi: 10.1371/journal.pone.0130464 (PMC4633155; doi:10.1371/journal.pone.0130464)
Supplement: S1 File — The ex vivo ELISPOT using PBMCs from 2 healthy individuals: HD-09 (Table A), HD-10 (Table B), do not show significant difference when incubated with HIV-A2 peptide and when peptide is omitted. T-test comparing the triplicate wells from irrelevant peptide (HIV-A2) control and no-peptide control (CM) of 2 healthy individuals showed p > 0.050 (Table C). (PDF) [file pone.0130464.s002.pdf]

S1 File

Table A

HD-09

	IFNg (spots/ 1e5 PBMCs)				GzmB (spots/ 1e5 PBMCs)			
	Well (1)	Well (2)	Well (3)	Avg	Well (1)	Well (2)	Well (3)	Avg
Pep-01	11	4	3	5.70	17	4	9	10.09
Pep-02	9	8	17	11.40	9	5	12	8.77
Pep-03	9	5	20	11.40	17	5	3	8.33
Pep-04	55	24	24	34.21	34	14	20	22.81
Pep-05	8	34	24	21.93	14	12	16	14.04
Pep-06	20	8	9	12.28	3	4	1	2.63
Pep-07	9	25	24	19.30	11	21	30	20.61
Pep-08	28	22	11	20.18	4	13	34	17.11
FluM	42	41	64	49.12	24	18	20	20.61
HIV-A2		13	9	11.18	1	18	12	10.53
No peptide	12	11	12	11.40	5	22	8	11.84

Table B

HD-10

	IFNg (spots/ 1e5 PBMCs)				GzmB (spots/ 1e5 PBMCs)			
	Well (1)	Well (2)	Well (3)	Avg	Well (1)	Well (2)	Well (3)	Avg
Pep-01	16	8	18	14.07	6	11	8	8.52
Pep-02	17	15	8	13.33	11	9	18	12.78
Pep-03	12	26	0	12.59	19	12	14	15.00
Pep-04	22	4	13	13.33	15	7	16	12.59
Pep-05	13	15	15	14.44	7	8	9	8.15
Pep-06	7	18	6	10.37	18	16	24	19.26
Pep-07	2	7	4	4.26	6	4	13	7.78
Pep-08	4	15	11	10.00	6	13	8	8.70
FluM	7		17	11.67	12	27	19	19.44
HIV-A2	6	11	10	9.07	7	17	14	12.78
No peptide	2	10	8	6.48	18	9	6	10.74

Table C

T-test comparing HIV-A2 and no peptide control

	IFNg	GzmB
HD-09	0.930	0.866
HD-10	0.434	0.687
